# The lived experiences of operating room nurses from the surgery on COVID-19 patients: a phenomenological study

**DOI:** 10.1186/s13741-024-00383-x

**Published:** 2024-04-24

**Authors:** Behzad Imani, Mehrnush Mostafayi, Shirdel Zandi

**Affiliations:** 1 Department of Operating Room, School of Paramedicine, Hamadan University of Medical Sciences, Hamadan, Iran; 2 Department of Anesthesia and Surgical Technology, School of Paramedicine, Urmia University of Medical Sciences, Urmia, Iran

**Keywords:** COVID-19, Lived experiences, Operating room, Perception, Phenomenology, Qualitative research

## Abstract

**Background:**

During the COVID-19 pandemic, some patients who were transported to the operating room for emergency surgery had COVID-19; operating room nurses should be in direct contact with these patients in a small and closed space of the operating room. This can lead to unpleasant experiences for these people. Accordingly, this study was conducted to understand the experience of operating room nurses during the surgery of COVID-19 patients.

**Methods:**

This qualitative study is a descriptive phenomenological study. Sampling was done purposefully and participants were selected based on the inclusion and exclusion criteria. The data of this study was obtained through semi-structured interviews with 12 participants and analyzed using the Colaizzi method.

**Results:**

Four main themes and 13 sub-themes were presented in this study: (1) feeling heroic (being a savior, self-sacrificing). (2) Exacerbating burnout (emotional exhaustion, feeling of incompetence, physical overtiredness). (3) Psychiatric crisis (destructive anxiety, horror of death, worrying about being a carrier, drastic feeling of pity). (4) Feeling the need for support (need for professional support, need for emotional support, need for social support).

**Conclusion:**

The results of this study show that operating room nurses experienced conflicting feelings during surgery on patients with COVID-19. So the feeling of being a hero was a heartwarming experience, but the aggravation of job burnout and mental crisis was unpleasant for them. Also, these people have experienced the need to be supported in various aspects.

## Introduction

The coronavirus disease 2019 (COVID-19) is a contagious respiratory tract disease caused by severe acute upper respiratory syndrome coronavirus-2 (SARS-CoV-2) (Guan et al. 2019). It appeared in Wuhan, China, in 2019 and spread rapidly worldwide, with the World Health Organization (WHO) classifying COVID-19 as a pandemic on March 11, 2020 (WHO 2020). According to WHO statistics, at the time of writing, about 510 million people have been infected worldwide, of which about 6.2 million have died. In Iran, about 7 million people have been infected, of which about 141,000 have died (WHO COVID-19 Dashboard 2020). In this century, this pandemic, which has a very high mortality rate, is one of the main threats to the health status of human beings, especially professional healthcare providers (Yang et al. 2020).

Healthcare providers are at the forefront of combating COVID-19 (Khaled et al. 2022). The WHO has considered these people one of the most vulnerable groups during this pandemic (Zhang et al. 2020). Despite the precautions taken and proper protection measures, healthcare providers are constantly threatened, so a high percentage of these people are infected, which causes the death of a large number of care providers (Bandyopadhyay et al. 2020). In this field, studies have shown that mental and emotional suffering can affect the performance and health of healthcare workers, which can threaten their safety and that of patients, this expresses the importance of knowing the traumatic experiences of these people (Resilience 2019). In the wake of the recent pandemic, various studies have examined the experiences of healthcare providers in delivering care to patients with COVID-19, which differ based on the type of expertise and the region under investigation (Karimi et al. 2020; Hantoushzadeh et al. 2021).

Like other parts of the hospital, the pandemic affected the operating room and disrupted its usual activity (Prakash et al. 2020). The elective surgeries were delayed or canceled during the Coronavirus pandemic (Lee et al. 2020). However, the operating room and its personnel still provide care in emergency cases (Simone et al. 2020; Li et al. 2020a). Even a positive COVID-19 patient should be transferred to the operating room for surgery in emergencies (Coccolini et al. 2020). As part of the surgical team, the operating room nurses in a small and closed space have close contact with COVID-19 patients, which has caused some of these personnel to be contaminated (McDougal et al. 2022). Various studies show the emergence of serious challenges such as the application of new care protocols and standards, professional challenges, and psychological crises in operating rooms with the outbreak of COVID-19 (Wong et al. 2020; Mohammadi et al. 2021). In Iran, no study has directly investigated the experiences of Non-physician members of surgical teams in order to identify their support and rehabilitation needs. As a result, this study was conducted to understand the experience of operating room nurses from the surgery on COVID-19 patients.

## Methods

### Study setting

A descriptive phenomenological approach was used in this qualitative inquiry. The philosophy of phenomenology of Husserl has been widely used to understand human phenomena as it is experienced or lived by individuals and involves direct exploration, analysis, and description of the said phenomena aiming at maximum intuitive presentation (Hassan 2023). This study has been prepared based on the consolidated criteria for reporting qualitative studies (COREQ) (Booth et al. 2014).

### Participants and sampling

Sampling was done purposefully and participants were selected based on inclusion criteria. In this purposeful sampling, participants were selected among those operating room nurses who had sufficient experience performing surgery on patients with COVID-19. The reason for purposive sampling is the better matching of the sample to the aims and objectives of the research, thus improving the rigor of the study and the trustworthiness of the data and results (Campbell et al. 2020). Before starting the interview, the purpose of the study was fully explained to the participants and the researcher provided her academic profile to the participants. After providing the necessary information about the study, the informed consent form was given to the participants and they were asked to read and sign it to express their agreement to start the interview. Also, all participants were made aware that they were free to withdraw from the study at any time or stage, without providing any reasons. All the interviews were conducted with the opinion of the participants, in the conference room of the operating room and during their work shifts. All interviews were conducted face-to-face and only with the presence of the researcher and the interviewee. The sample size was not determined at the beginning of the study, but the sampling continued until data saturation. Participants were selected from operating room nurses who worked in the operating room of Besat Hospital in Hamadan-Iran during the COVID-19 pandemic. Participants were purposefully selected based on the following inclusion criteria: (1) a non-physician member of the surgical team, (2) interest in participating in the research and expressing their experiences, (3) no confirmed psychiatric illness, (4) enough experience in providing emergent surgery to COVID-19 patients during the COVID-19 pandemic in an operating room. Furthermore, individuals were excluded from the study under the following conditions: (1) inability to effectively convey their experiences, (2) refusal to answer the interviewer’s questions, and (3) unwillingness to continue participation.

### Data collection

Before starting the interview, the demographic characteristics of the participants were recorded using a checklist. The interviews began with a non-structured question (tell us about your experience of participating in performing the surgery for COVID-19 patients) and continued with semi-structured questions. At the beginning of the study, two interviews were conducted as a pilot and the data were critically evaluated so that the interview process and questions could be modified if necessary. Each interview lasted 35–70 min and was conducted in two sessions if necessary that in two cases, due to the need to clarify the data and understand the participant's intention, the interview was repeated. All interviews were conducted by the leading investigator (ShZ), who has experience in qualitative research and interviewing. The interview was recorded and then written down with the participant's permission. In order to confirm the codes extracted from each interview, the analysis results were provided to the participants. The next interviewee was chosen based on the data analysis outcomes from prior interviews. Sampling continued until the data was saturated so that new data and information could not be extracted from the interviews.

### Data analysis

The descriptive Colaizzi method was used to analyze the collected data (Colaizzi 1978). This method consists of seven steps: (1) collecting the participants’ descriptions, (2) understanding the meanings in depth, (3) extracting important sentences, (4) conceptualizing important themes, (5) categorizing the concepts and topics, (6) constructing comprehensive descriptions of the issues examined, and (7) validating the data following the four criteria set out by Lincoln and Guba. In this regard, the interview texts were read independently and repeatedly by two researchers (ShZ, MM) for the first time. In this way, the data were examined, and an attempt was made to understand what was being explained. Significant statements in the interview texts were selected and generally expressed. Then, the implicit data within the statements were identified and analyzed. The researchers formulated the meanings by discussing them until a consensus was reached and they were validated. Subsequently, themes were identified and organized into clusters and categories. The research themes and subcategories were developed with a clear statement. The research findings were presented to the participants, and the accuracy of the themes and content was strengthened. In addition, the participants’ opinions were referenced so that the reader could confirm the interpretation and analysis of the data. MAXQDA software version 10 was used for data management during the data analysis process.

### Rigor

Trustworthiness criteria were used to validate the research since data and findings validity were important in qualitative research (Creswell 2002). This study was based on four criteria of Lincoln and Guba: credibility, transferability, dependability, and conformability (Northcote 2012). For data credibility, prolonged engagement and follow-up observations, as well as samplings with maximum variability were used. For the dependability of the data, the researchers were divided into two groups, and the research was conducted as two separate studies to evaluate the sameness of the results of the two groups. At the same time, another researcher with the most familiarity and ability in conducting qualitative research supervised the study as an external observer. Concerning conformability, the researchers tried not to influence their own opinions in the coding process. Moreover, the codes were read out by the participants as well as two researcher colleagues with the help of an independent researcher and expert familiar with qualitative research. The transferability of data was confirmed by offering a comprehensive description of the subject, participants, data collection, and data analysis.

## Results

Twelve operating room nurses participated in this study. The participants’ mean age and work experience were 34.41 and 9.58 years, respectively. 41.66% of the participants were female and 66.66% were married. Of these, 2 were masters of surgical technology and 10 had a bachelor of surgical technology (Table 1).

**Table 1:** The demographic characteristics of participants

Participants	Education	Work experience (year)
P1	Bachelor of Surgical Technology	14
P2	Bachelor of Surgical Technology	16
P3	Master of Surgical Technology	12
P4	Bachelor of Surgical Technology	11
P5	Bachelor of Surgical Technology	17
P6	Bachelor of Surgical Technology	2
P7	Bachelor of Surgical Technology	7
P8	Bachelor of Surgical Technology	3
P9	Bachelor of Surgical Technology	5
P10	Bachelor of Surgical Technology	15
P11	Master of Surgical Technology	4
P12	Bachelor of Surgical Technology	9

Analysis of the surgical technology experiences of performing the surgery on COVID-19 patients by descriptive phenomenology revealed four main themes: feeling heroic, exacerbating burnout, psychiatric crisis, and feeling the need for support (Table 2).

**Table 2:** The themes and sub-themes

Themes		Sub-themes
1	Feeling heroic	Being a savior
		Self-sacrificing
2	Exacerbating burnout	Emotional exhaustion
		Feeling of incompetence
		Physical overtiredness
3	Psychiatric crisis	Destructive anxiety
		Horror of death
		Worrying about being a carrier
		Drastic feeling of pity
4	Feeling the need to support	Need for professional support
		Need for emotional support
		Need for social support

### Feeling heroic

The experience of operating room nurses shows that these people have experienced positive emotions and pride after joining the surgical team of a patient with COVID-19. These people have felt like heroes because they have played a direct role on the battlefield with this highly contagious disease. In fact, because he is trying to help another person to survive, he thinks of himself as a hero. This feeling is manifested in two sub-themes being a savior and self-sacrificing.

#### Being a savior

Explaining the participants' lived experiences shows that while providing care and treatment services in the operating room to patients with COVID-19, they considered patients as victims whose lives were saved by the surgical team. “…I have played a role in this dangerous situation as a medical staff member. Despite all the dangers that threatened me and those around me, adhering to my responsibilities and standing firm against this disease, I feel like a savior for these patients …” (P3).

#### Self-sacrificing

The experience of operating room nurses shows that when these people are present in the surgical team of a patient with COVID-19, they consider playing this role beyond their duty. And these people have expressed with different statements that adhering to their responsibilities in this dangerous situation is a sign of their self-sacrifice. “…When a person enters a battlefield to save the life of a soldier who has been shot, he may also be wounded, which is a sign of that person's self-sacrifice… Our treatment measures in the closed environment of the operating room for the patient with COVID-19 reflect the same phenomenon…” (P7).

### Exacerbating burnout

The lived experience of operating room nurses conveys the message that these individuals have experienced an exacerbation of burnout during the COVID-19 pandemic. The long-term exposure of operating room nurses to the problematic conditions that this pandemic created for healthcare providers has led to an exacerbation of burnout among operating room nurses. In fact, the risk factors of extreme workload, extreme stress, role tension, and lack of time for recovery have caused the burnout of these people to intensify. This phenomenon in operating room nurses is due to emotional exhaustion, feelings of incompetence, and physical overtiredness.

#### Emotional exhaustion

Operating room nurses have been exposed to severe job stress from the COVID-19 pandemic for a relatively long time. The mentioned stresses have been a wide range of destructive phenomena that have left a synergistic effect on the mental state of individuals. According to the analysis, these tensions have created a wide range of emotional exhaustion for operating room nurses. “… During the third peak of the COVID-19 pandemic, I was under so much pressure and mental tension that I was helpless, and I was numb like a robot. I felt like I was stuck in this situation, and I had no choice…” (P10).

#### Feeling of incompetence

The specific conditions of this disease (ignorance, high transmission rate, recurrence rate, lack of decisive treatment, etc.) caused operating room nurses to find their efforts useless in the stages of their fight against this disease and experience a sense of inefficiency. “…At one time, I was disappointed in our efforts; we tried our best, but the number of patients was increasing every day; I felt that our efforts were in vain …” (P2).

#### Physical overtiredness

The lived experience of operating room nurses indicates that these people have become doubly tired due to the work pressures caused by a large number of patients and the constant and long-term use of PPE. “…Think about it; we had to carry this personal protective equipment (PPE) with us from the beginning to the end of the shift. Each shift, we went for a few minor and major operations. This situation lasted so long that I felt that my body was about to disintegrate one day…” (P6).

### Psychiatric crisis

Explaining the lived experience of operating room nurses shows that these people were under severe psychological stress during the COVID-19 pandemic. According to the nurses in the operating room, this psychological pressure was experienced in a very intense way and happened in a short period of time, and its scope was so comprehensive and intense that it can be considered a psychological crisis. So many people have said that they have never been affected by such intense mental pressure. A psychological crisis is manifested by the phenomena of destructive anxiety, horror of death, worrying about being a carrier, and feeling of drastic feelings of pity.

#### Destructive anxiety

The lived experience of operating room nurses shows the emergence of dimensions of destructive anxiety in these people during the COVID-19 pandemic, which has created an unpleasant experience for them. These people have stated that the destructive anxiety caused by the COVID-19 pandemic in these people has been so destructive and harmful that it has been able to significantly affect the quality of care and quality of life in various dimensions. “…I was always calm at work and in life; after a few months from the beginning of the pandemic and the peak of the disease, I was under so much stress that it made me an utterly anxious technologist, so I realized myself dimensions of an anxious life…”(P11).

#### Horror of death

Explaining the lived experience of operating room nurses conveys the message that these people have been a horror of death at various times during this pandemic. In fact, according to these people, factors such as the strength and rate of deaths from the disease and close exposure to it have caused these people to experience the horror of the possibility of death for themselves and those around them. “…A few days after the death of our colleague due to Corona, when I was operating on a COVID-19 patient, a series of unpleasant thoughts came to my mind that made me experience the horror of death, I imagined myself in the clutches of this killer virus that could kill me at any moment…” (P2).

#### Worrying about being a carrier

According to the lived experience of operating room nurses, one of the most unpleasant experiences for these people is worrying about being a carrier. These people have stated that the thought of being a carrier and making their family and relatives sick has always caused them to experience a worrying about being a carrier in this regard. “…my wife was pregnant; during this critical period of his life, instead of me being by his side all the time and providing him with safe conditions … I was always coming back from work when I was in contact with several people with COVID-19 … that was why I was constantly worried about infecting my wife…” (P1).

#### Drastic feeling of pity

Explaining the lived experience of operating room nurses shows that these people have experienced a strong sense of pity for patients with COVID-19 who have had severe disease. As these people have stated, seeing people with severe respiratory distress has caused them to feel pity and discomfort.“…The patient was a middle-aged man who was suffering from respiratory distress… I was heartbroken when I saw him breathing, and I was heartsick that I could not do anything for him …” (P5).

### Feeling the need to support

Analyzes of the living experience of operating room nurses have shown that these individuals need some outside support while providing care in the fight against the COVID-19 pandemic. In fact, according to the lived experience of these people, factors such as high workload, anxiety, cross-sectional occupational hazards, high risk of infection, etc., have caused these people to feel the need to receive exceptional support to survive. The support needs of these people have three sub-themes: the need for professional support, the need for emotional support, and the need for social support.

#### Need for professional support

The experience of operating room nurses indicates the need for professional support. These people have stated that in the fight against COVID-19, there is a need for PPE, sufficient human resources, up-to-date care facilities, etc. The organization must provide infrastructure and supplies for these people. “…by trusting in this PPE, we put ourselves in direct contact with positive COVID-19 patients… therefore, we need the organization to provide us with the best and most reliable equipment.…” (P10).

#### Need for emotional support

The experience of operating room nurses shows that these people have been emotionally traumatized by being on the battlefield for a long time and enduring extreme pressures. accordingly, these individuals have felt the need for emotional support while providing care. “… I had taken this disease home with me from work, my old parents were infected, and they were not in good condition, and I was under a lot of pressure in terms of employment… All this made me feel the need to see a counselor…” (P4).

#### Need for social support

Explaining the lived experience of operating room nurses shows that these people have also experienced the need for social support. According to them, because operating room nurses are waging a fierce and breathtaking struggle to support all members of society, the pandemic has placed social constraints on them. Accordingly, these people have experienced the need for social support. “…my colleagues and I stood by the people in these difficult and critical situations and devoted a considerable part of our social life to the care needs of COVID-19 patients … far beyond our means, we met the needs and expectations of the community … that is why I expect society to support us in this way as much as possible.…” (P12).

## Discussion

This qualitative study explores the lived experiences of operating room nurses from the surgery on COVID-19 patients. Based on the analysis of the experiences of operating room nurses, four main themes of feeling heroic, exacerbating burnout, psychiatric crisis, and the feeling of the need for support were identified, each consisting of several sub-themes.

This study showed that operating room nurses experienced a sense of heroism in performing surgery on a patient with COVID-19. In this regard, in their study, Oh Hee and Na Kyoung 2021 stated that nurses, with the help of patients with COVID-19, have felt proud of their field (Oh and Lee 2021). Explaining the lived experience of operating room nurses shows that the feeling of heroism in these people is created by the two sub-themes of being a savior and self-sacrificing. Being a savior is a pleasant experience that operating room nurses have found by performing surgery on patients with COVID-19. A study by Ponnambily Chandy et al. 2020, found that nurses were proud to have been able to save patients’ lives with COVID-19 (Chandy et al. 2022). As described in the study by ShanMohammed et al. 2021, nurses were described as having the necessary sacrifice in the fight against COVID-19 (Mohammed et al. 2021), our study also showed that operating room nurses experienced a sense of self-sacrificing during the fight against COVID-19.

According to the results of this study, another experience that operating room nurses have gained during surgery on patients with COVID-19 has been exacerbating burnout. Various studies have shown that the COVID-19 pandemic has brought different dimensions of burnout to care providers (Rahmani et al. 2021; Hoseinabadi et al. 2020). Severe burnout, existing stress, complex patient care, unclear disease status, and system inefficiency can affect the quality of nursing care (Hamers et al. 2016). According to the lived experience of operating room nurses, the phenomenon of exacerbating burnout is manifested in the three sub-themes of emotional exhaustion, feeling of incompetence, and physical overtiredness. Emotional exhaustion in operating room nurses is an unpleasant experience that can result from being under job stress. In this regard, the Hansol Hwang 2020 study has shown that emotional exhaustion among the workforce in various fields has increased significantly during the COVID-19 pandemic (Hwang et al. 2021). Analyzes also showed that operating room nurses experienced an unpleasant feeling of incompetence in the face of patients with severe pulmonary involvement because they believed they could not help the patient. Nursing has always sought to provide effective services and care. In this regard, the study of Galehdar et al. 2020 has shown that when patients suffer from respiratory distress and the nurse cannot do anything for them; it has many adverse effects on their mood (Galehdar et al. 2021). Another experience related to burnout in operating room nurses during the COVID-19 pandemic is the issue of physical overtiredness in these individuals. This experience in operating room nurses is due to the high workload and permanent and long-term use of PPE. In this regard, the study of Hyunjie Lee 2022 et al. showed that nurses in the field of COVID-19 had experienced severe physical fatigue due to PPE (Lee et al. 2022). Unusually physical overtiredness as a result of these themes must be countered with self-care to allow nurses to continue to provide high-quality, genuine patient care (Xie et al. 2020).

The psychiatric crisis is another unpleasant phenomenon known in this study that operating room nurses have experienced from surgery on patients with positive COVID-19. In this regard, it has been shown that healthcare providers experienced many psychological disorders such as depression, anxiety, insomnia, and stress, while female nurses and nurses who were on the front lines of the fight against COVID-19 had more severe psychological problems (Li et al. 2020b). The psychiatric crisis of operating room nurses will be manifested in four sub-themes: destructive anxiety, horror of death, worrying about being a carrier, and feeling of drastic feelings of pity. The well-known destructive anxiety of operating room nurses during this pandemic was, in fact, their mental reaction to stressful situations. In this regard, previous studies have identified anxiety as a psychological response in care providers during the COVID-19 pandemic (Fernandez et al. 2021; Salari et al. 2020). In such a stressful situation, nurses need intelligent resilience to provide higher-quality care measures (Imani et al. 2018). Another psychological stress experienced by operating room nurses is the horror of death. The horror of death experienced by these people was in response to the high prevalence and mortality rate of the disease. It has been shown that one of the most important reasons for the fear and worry of nurses is the fear of getting infected, and in this regard, the safety infrastructure should be strengthened so that nurses do not have to worry about anything other than patient care (Galehdar et al. 2021). in this regard, the study of Karimi et al. 2020 has shown that fear of death has become a challenge that distracts nurses from genuine patient care (Karimi et al. 2020). Another unpleasant experience identified is worrying about being a carrier, as shown in the study by Didem Coşkun Şimşek RN et al. 2021, this feeling of worrying about being a carrier is the fear of infection and transmission of the disease to one’s family (Coşkun Şimşek and Günay 2021). Another experience of operating room nurses is a drastic feeling of pity. In this regard, the study by Martins-Akinlose et al. 2021 has shown that this disease, in addition to patients and their families, can also cause inconvenience and confusion to care providers (Martins-Akinlose et al. 2021).

Another finding of this study is the need for the support of operating room nurses. Previous studies have shown that employees in their workplace need support in different dimensions (Imani et al. 2014, 2022). According to the analysis, this phenomenon consists of three sub-themes: the need for professional support, the need for emotional support, and the need for social support. From the operating room nurses’ point of view, the need for professional support during this pandemic is the provision of infrastructure and standard equipment and facilities. In this regard, a study by Hyunjie Lee et al. 2020 showed that a poor work environment has made volunteer nurses feel uncomfortable in the face of COVID-19 (Lee et al. 2022). In this situation, the level of managers’ professional skills can significantly affect nurses’ stress levels (Hamidi et al. 2012). Due to the double physical and mental stress during this pandemic, operating room nurses have stated that they have experienced the need for emotional support. In this regard, previous studies have also proved the need for emotional support for the treatment cadre (Frias et al. 2020; Miotto et al. 2020). Another perceived supportive need of operating room nurses is the need for social support during this pandemic. Operating room nurses have experienced the need to have people's attention in the community. In this regard, Ponnambily Chandy et al. 2020 showed that healthcare providers need to be supported by the community such as praise, respect, and recognition (Chandy et al. 2022).

## Limitations

However, the current study has a series of limitations, some of which are specific to qualitative studies. First, operating room nurses participating in this study were selected from a hospital, considering that the level of hospital equipment and facilities can affect the work experience of employees, it is possible that people working in other centers may have different experiences that could not be identified in this study. Another limitation of this research is data collection in emergency conditions and in the shortest possible time, which limited this time for better data validation.

## Conclusion

The study provided a comprehensive and in-depth understanding of the lived experiences of operating room nurses from the surgery on COVID-19 patients through a phenomenological study. From the findings of this study, it can be concluded that operating room nurses by surgery on positive COVID-19 patients, other than feeling heroic, have mainly had unpleasant experiences that include: exacerbating burnout, psychiatric crisis, and the feeling of the need for support. In addition to endangering patient safety, this can lower the standard of surgical care provided in the operating room. Accordingly, healthcare systems must implement the necessary support measures for operating room nurses to preserve the health of operating room nurses and the safety of surgery candidate patients. This study’s results suggest that to prepare for the crisis of new epidemics, operating rooms should have dedicated sections for admitting patients with contagious respiratory diseases to reduce the risk of disease transmission in the operating room’s limited space.

## Implications for practice

Paying attention to the identified experiences and applying measures that can improve the working conditions of operating room nurses can directly and indirectly improve the items of work quality, quality of care, and job satisfaction.

## Data Availability

The datasets used and/or analyzed during the current study are available from the corresponding author on reasonable request.

## References

[CR1] Bandyopadhyay S, Baticulon RE, Kadhum M, Alser M, Ojuka DK, Badereddin Y (2020). Infection and mortality of healthcare workers worldwide from COVID-19: a systematic review. BMJ Glob Health.

[CR2] Booth A, Hannes K, Harden A, Noyes J, Harris J, Tong A (2014). COREQ (consolidated criteria for reporting qualitative studies). A user's manual Guidelines for reporting health research.

[CR3] Campbell S, Greenwood M, Prior S, Shearer T, Walkem K, Young S (2020). Purposive sampling: complex or simple? Research case examples. J Res Nurs.

[CR4] Chandy P, Kanthi E, Pradeep P, Sathianathan P, Jebakamal S, Narchaithi M (2022). Lived experience of health-care providers during COVID-19: a meta-synthesis. Indian J Psychiatry.

[CR5] Coccolini F, Perrone G, Chiarugi M, Di Marzo F, Ansaloni L, Scandroglio I (2020). Surgery in COVID-19 patients: operational directives. World J Emerg Surg.

[CR6] Colaizzi PF. Psychological research as the phenomenologist views it. 1978.

[CR7] Coşkun Şimşek D, Günay U (2021). Experiences of nurses who have children when caring for COVID-19 patients. Int Nurs Rev.

[CR8] Creswell JW (2002). Educational research: planning, conducting, and evaluating quantitative.

[CR9] De Simone B, Chouillard E, Di Saverio S, Pagani L, Sartelli M, Biffl W (2020). Emergency surgery during the COVID-19 pandemic: what you need to know for practice. Ann R Coll Surg Engl.

[CR10] Fernandez R, Lord H, Moxham L, Middleton R, Halcomb E (2021). Anxiety among Australian nurses during COVID-19. Collegian (Royal College of Nursing, Australia).

[CR11] Frias CE, Cuzco C, Martín CF, Pérez-Ortega S, Triviño López JA, Lombraña M (2020). Resilience and emotional support in health care professionals during the COVID-19 pandemic. J Psychosoc Nurs Ment Health Serv.

[CR12] Galehdar N, Toulabi T, Kamran A, Heydari H (2021). Exploring nurses' perception of taking care of patients with coronavirus disease (COVID-19): a qualitative study. Nurs Open.

[CR13] Guan W-j, Ni Z-y, Hu Y, Liang W-h, Ou C-Q, He J-x (2020). Clinical characteristics of coronavirus disease 2019 in China. N Engl J Med..

[CR14] Hamers J, Nijhuis-van der Sanden M, Ettema R, Heinen M, Huisman-de Waal G, de Man-van Ginkel J (2016). Essential nursing care: most provided, least evidence based. The basic care revisited program. J Adv Nurs..

[CR15] Hamidi Y, Mehri M, Zamanparvar A, Imani B (2012). Relationship between managerial skills and employees job stress in health centers. J Res Health Sci.

[CR16] Hantoushzadeh S, Bagheri M, Amjadi MA, Farahani MF, Haghollahi F (2021). Experiences of health care providers on pregnancy and childbirth care during the COVID-19 pandemic in Iran: a phenomenological study. BMC Pregnancy Childbirth.

[CR17] Hassan MA-S. The use of Husserl's phenomenology in nursing research: a discussion paper. J Adv Nurs. 2023. 10.1111/jan.15564 36718849

[CR18] Hwang H, Hur WM, Shin Y (2021). Emotional exhaustion among the South Korean workforce before and after COVID-19. Psychol Psychother Theory Res Pract.

[CR19] Imani B, Karamporian A, Hamidi Y (2014). The relationship between quality of work life and job stress in employees the foundation of martyrs and veterans affairs of Hamadan. J Mil Med.

[CR20] Imani B, Kermanshahi SMK, Vanaki Z, Kazemnejad LA (2018). Hospital nurses’ lived experiences of intelligent resilience: a phenomenological study. J Clin Nurs.

[CR21] Imani B, Zandi S, Mostafayi M, Zandi F (2022). Presentation of a model of the work engagement in surgical technologists: a qualitative study. Perioper Care Oper Room Manag.

[CR22] Karimi Z, Fereidouni Z, Behnammoghadam M, Alimohammadi N, Mousavizadeh A, Salehi T (2020). The lived experience of nurses caring for patients with COVID-19 in Iran: a phenomenological study. Risk Manag Healthc Policy.

[CR23] Khaled MFI, Sobhan SMM, Chowdhury MT, Hossain MK, Ahmed S, Chowdhury MM (2022). Perceptions and behaviors of forefront healthcare fighters about COVID-19 during its pandemic situation in Bangladesh-a rapid survey. KYAMC J.

[CR24] Lee J, Choi J, Kim M (2020). Elective surgeries during the COVID-19 outbreak. J Br Surg..

[CR25] Lee H, Lee SE, Sang S, Morse B. The lived experience of nurses who volunteered to combat the COVID‐19 pandemic in South Korea: a qualitative phenomenological study. J Nurs Manag. 2022. 10.1111/jonm.13571 PMC9115118 35229395

[CR26] Li Y, Zeng L, Li Z, Mao Q, Liu D, Zhang L (2020). Emergency trauma care during the outbreak of corona virus disease 2019 (COVID-19) in China. World J Emerg Surg.

[CR27] Li W, Yang Y, Liu Z-H, Zhao Y-J, Zhang Q, Zhang L (2020). Progression of mental health services during the COVID-19 outbreak in China. Int J Biol Sci.

[CR28] Martins-Akinlose OD, Aluko D, Joel O. Lived experience and quality of life of frontline health care workers caring for patients with Covid-19 in Lagos State. 2021.

[CR29] McDougal AN, Elhassani D, DeMaet MA, Shores S, Plante KS, Plante JA (2022). Outbreak of coronavirus disease 2019 (COVID-19) among operating room staff of a tertiary referral center: An epidemiologic and environmental investigation. Infect Control Hosp Epidemiology.

[CR30] Miotto K, Sanford J, Brymer MJ, Bursch B, Pynoos RS (2020). Implementing an emotional support and mental health response plan for healthcare workers during the COVID-19 pandemic. Psychol Trauma.

[CR31] Mohammadi F, Tehranineshat B, Bijani M, Oshvandi K, Badiyepeymaiejahromi Z (2021). Exploring the experiences of operating room health care professionals' from the challenges of the COVID-19 pandemic. BMC Surg.

[CR32] Mohammed S, Peter E, Killackey T, Maciver J (2021). The, “nurse as hero” discourse in the COVID-19 pandemic: a poststructural discourse analysis. Int J Nurs Stud.

[CR33] Northcote MT. Selecting criteria to evaluate qualitative research. 2012.

[CR34] Oh H, Lee NK (2021). A phenomenological study of the lived experience of nurses caring for patients with COVID-19 in Korea. J Korean Acad Nurs.

[CR35] Prakash L, Dhar SA, Mushtaq M (2020). COVID-19 in the operating room: a review of evolving safety protocols. Patient Saf Surg.

[CR36] Rahmani R, Sargazi V, Jalali MS, Babamiri M (2021). Relationship between COVID-19-caused anxiety and job burnout among hospital staff: a cross-sectional study in the Southeast of Iran. J Occup Hyg Eng.

[CR37] Resilience M. Moral Resilience: Transforming Moral Suffering in Healthcare2019. 2019;39(1).

[CR38] Salari N, Khazaie H, Hosseinian-Far A, Khaledi-Paveh B, Kazeminia M, Mohammadi M (2020). The prevalence of stress, anxiety and depression within front-line healthcare workers caring for COVID-19 patients: a systematic review and meta-regression. Human Resour Health.

[CR39] Sarboozi Hoseinabadi T, Kakhki S, Teimori G, Nayyeri S. Burnout and its influencing factors between frontline nurses and nurses from other wards during the outbreak of Coronavirus Disease -COVID-19- in Iran. Invest Educ Enferm. 2020;38(2):e3. 10.17533/udea.iee.v38n2e03. 10.17533/udea.iee.v38n2e03 PMC7883923 33047546

[CR40] WHO. WHO announces COVID-19 outbreak a pandemic 2020 [Available from: https://www.euro.who.int/en/health-topics/healthemergencies/coronavirus-covid-19/news/news/2020/3/whoannounces-covid-19-outbreak-a-pandemic.

[CR41] WHO COVID-19 Dashboard. Geneva: World Health Organization, 2020. Available online: [cited 2022 May 2,]. Available from: https://covid19.who.int/.

[CR42] Wong J, Goh QY, Tan Z, Lie SA, Tay YC, Ng SY (2020). Preparing for a COVID-19 pandemic: a review of operating room outbreak response measures in a large tertiary hospital in Singapore. Can J Anaesth.

[CR43] Xie J, Tong Z, Guan X, Du B, Qiu H, Slutsky AS (2020). Critical care crisis and some recommendations during the COVID-19 epidemic in China. Intensive Care Med.

[CR44] Yang X, Yu Y, Xu J, Shu H, Liu H, Wu Y (2020). Clinical course and outcomes of critically ill patients with SARS-CoV-2 pneumonia in Wuhan, China: a single-centered, retrospective, observational study. Lancet Respir Med.

[CR45] Zhang SX, Liu J, Jahanshahi AA, Nawaser K, Yousefi A, Li J (2020). At the height of the storm: healthcare staff’s health conditions and job satisfaction and their associated predictors during the epidemic peak of COVID-19. Brain Behav Immun.

